# Health problems in elderly patients during the first post-stroke year

**DOI:** 10.3109/03009734.2012.674572

**Published:** 2012-08

**Authors:** Lena Olai, Lars Borgquist, Kurt Svärdsudd

**Affiliations:** ^1^Department of Public Health and Caring Sciences, Uppsala University, Family Medicine and Preventive Medicine Section; ^2^Centre for Clinical Research, Dalarna, Sweden; ^3^Dalarna University, School of Health and Social Studies, Falun, Sweden; ^4^Linköping University, Medical and Health Sciences, Linköping, Sweden

**Keywords:** Documentation, health problem, nursing, prevalence, time course

## Abstract

**Background:**

A wide range of health problems has been reported in elderly post-stroke patients.

**Aim:**

The aim of this study was to analyse the prevalence and timing of health problems identified by patient interviews and scrutiny of primary health care and municipality elderly health care records during the first post-stroke year.

**Methods:**

A total of 390 consecutive patients, ≥65 years, discharged alive from hospital after a stroke event, were followed for 1 year post-admission. Information on the health care situation during the first post-stroke year was obtained from primary health care and municipal elderly health care records and through interviews with the stroke survivors, at 1 week after discharge, and 3 and 12 months after hospital admission.

**Results:**

More than 90% had some health problem at some time during the year, while based on patient record data only 4–8% had problems during a given week. The prevalence of interview-based health problems was generally higher than record-based prevalence, and the ranking order was moderately different. The most frequently interview-reported problems were associated with perception, activity, and tiredness, while the most common record-based findings indicated pain, bladder and bowel function, and breathing and circulation problems. There was co-occurrence between some problems, such as those relating to cognition, activity, and tiredness.

**Conclusions:**

Almost all patients had a health problem during the year, but few occurred in a given week. Cognitive and communication problems were more common in interview data than record data. Co-occurrence may be used to identify subtle health problems.

## Introduction

It is well known that post-stroke patients have health problems ranging from minor difficulties to fatal events. Even in patients deemed to have recovered 3 months after a stroke, functional abilities and quality of life may still be impaired (1). Survivors of a mild stroke may have some degree of disability and need to change their life-styles for up to 1 year after the stroke incident (2). Other stroke survivors may have to cope with serious permanent cognitive decline (3).

Previous studies have indicated a wide range of health problems after a stroke incident, including pain, fever, infections, falls, depression, anxiety, emotionalism, confusion, fatigue, etc. (4–12). Most of these studies covered hospital patients; only a few covered nursing home patients and post-stroke patients living at home. This selection of study population may have caused higher health problem prevalence than would have been found in a more representative post-stroke population. Moreover, health problem assessments in these post-stroke patients were usually based on clinical examinations with a neurological focus. We have not found any studies with a broader, longitudinal approach where the change of health situation can be assessed over time.

This study was designed as a comprehensive project, where a fairly large and representative sample of stroke patients was followed during 1 year after the stroke event. In a previous report the ability of hospital-based nurses, physicians, physiotherapists, and occupational therapists to give a correct prognosis assessment regarding these patients was tested (13), and in another report survival, hazard function for a new event, and health care utilization among these patients were analysed (14).

In this report the prevalence of health problems in a broad sense during the first stroke year was measured, based on data from patient interviews, data from primary health care records, and patient records from assisted accommodation and nursing homes. The aim of the present study was to analyse the prevalence and timing of health problems identified through patient interviews and scrutiny of primary health care and municipality elderly health care records during the first post-stroke year.

## Study population and methods

### Setting

The study was performed in the cities of Falun (population 55,000) and Borlänge (population 47,000), central Sweden, with similar age and sex distributions as the Swedish national population (15). Both cities are served by Falun General Hospital, the only hospital in the area. Since admission to hospital in Sweden is free of charge for the patient, virtually all patients with clinical signs and symptoms indicating stroke are admitted (except some already institutionalized) (16). Moreover, patient fees for hospital out-patient clinics, appointments with general practitioners, and municipal support are all heavily subsidized by central and local governments, which means that private financial resources are seldom an obstacle to health care utilization.

All Swedish residents have a unique 12-digit personal identification number (PIN), given at birth or immigration and used in all official documents and registers. The PIN is an excellent and highly reliable tool for record linkage and retrieval.

### Study population

During the period 1 September 1999 to 31 May 2001 a total of 432 patients 65 years of age or older, living at home before admission and having no pre-admission dementia diagnosis, were cared for on an in-patient basis at the Department of Internal Medicine (stroke unit or general ward) after an acute stroke (index admission), defined as intracerebral haemorrhage, brain infarction, or stroke of undetermined pathological type (ICD10 codes I61, I63, and I64) (17). Forty-two potential participants died at the Department of Internal Medicine, and the 390 survivors constitute the study population of this report.

### Data collection

**Figure 1.**
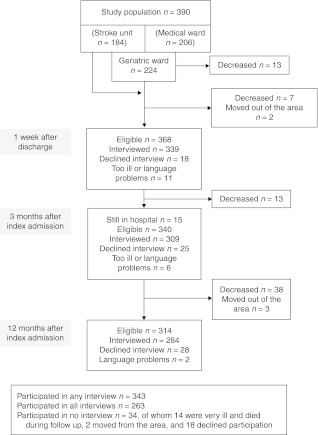
Flow chart of the study population.

Figure 1 shows the patient flow through the study. One week after discharge there was a standardized face-to-face interview with the patients in their homes, assisted accommodations, or nursing homes, repeated at 3 and 12 months after admission by two registered nurses. In case of aphasia or cognitive problems a next of kin was asked to participate to help interpret answers. Overall, 93% of the eligible patients were interviewed at least once. Forty interviews were performed in duplicate independently by the two observers with excellent agreement (kappa ≥ 0.95). Moreover, all primary health patient records and all municipal elderly health care nursing records regarding the study population were scrutinized.

#### Interviews

In the patient interviews information was sought on education, marital status, cohabitation, functional ability, Mini Mental State Examination (MMSE) (18), Hospital Anxiety and Depression Scale (HAD) (19), The Nottingham Health Profile (NHP) (20), self-reported health, and health problems. Functional ability was estimated using a Katz Activity of Daily Living (ADL) assessment, ranging from completely independent, grade A (= 1) to completely dependent, grade G (= 7) (21). All instruments are validity- and reliability-tested with good results. The part of the interview focused on medical or nursing problems was based on a pre-prepared form made for this study.

The HAD scale has 14 items, 7 on anxiety and 7 on depression, each with a four-point verbal rating scale scored 0–3, giving total scores ranging from 0 to 21. Scores of 8–10 on a subscale have been claimed to indicate possible pathology, and scores ≥ 11 as ‘definite cases’ (19). The NHP questionnaire, tested in the general population and in various patient populations, including stroke patients, measures discomfort (20). Part I, used in this study, consists of 38 yes/no questions forming six dimensions: energy (3 statements), physical mobility (8 statements), sleep (5 statements), emotional reactions (9 statements), social isolation (5 statements), and pain (8 statements). Statements were weighed within each area, resulting in scores ranging from 0 to 100, with 0 indicating no problems.

The participants were asked to grade their self-reported health on a five-degree ordinal scale, ranging from poor (= 1) to excellent (= 5) and to grade their sense of loneliness, ranging from never (= 0) to several times a week (= 3). Health problems were measured with a list of 30 medical or nursing problems and an indefinite number of open alternatives. The patients were asked which of these or other health problems they had experienced after the stroke or after the previous interview and whether the symptom was new or exacerbated. Possible responses were ‘yes’ or ‘no’.

#### Patient record scrutiny

Information on appointments with and home calls from the primary health care centre (PHCC) staff (all caregiver categories) and municipal elderly health care support was obtained by scrutiny of PHCC and municipal elderly health care records regarding health problems, diagnoses, and date of appointment or care given. The mean number of PHCC notes during the follow-up year was 41.5 (median 23, interquartile range 10–48), and the mean number of municipal record notes was 135.3 (median 124, interquartile range 57–205). All recorded problems were registered. No distinction was made between the concepts of complications, sequels, or stroke-related disability, to avoid classification uncertainty.

The health problems documented during the interviews and the diagnoses and health problems found after patient record scrutiny were classified into key words, each coded as yes/no, according to the Well-being, Integrity, Prevention, and Safety (VIPS) classification for nursing documentation in patient records (22). The VIPS key words and their contents are presented in Table I. The information on health problems based on interview data was available at 1 week after discharge from hospital and at 3 and 12 months after hospital admission, whereas the information based on patient record scrutiny was available continuously during the first post-stroke year. To facilitate comparison of the two ways of measuring health care problems the patient record-based data were computed with the same time-frame as the interview data in addition to the continuous mode.

**Table I.** Key words and their content in the Well-being, Integrity, Prevention, and Safety (VIPS) classification for nursing documentation.

Key word	Content
Communication	Speech impairment, aphasia, apraxia
Cognition, development	Memory deficits, concentration difficulty, understanding of health and illness, lack of initiative or motivation, attention impairment, difficulties with planning and organizing, post-stroke dementia
Breathing, circulation	Respiratory problems, aspiration, dyspnoea, coughing, heart problems, deep vein thrombosis, bleeding, body temperature, oedema, cyanosis
Nutrition	Swallowing difficulties, nausea, vomiting, appetite loss, weight loss
Elimination	Urinary tract problem, bladder or bowel incontinence, diarrhoea, constipation
Skin	Integument, lesions, ulcers, wound infection, sweating
Activity	Paralysis, falls, spasticity, contracture, deteriorated condition, activity limitations
Sleep	Fatigue, day-time sleepiness, tiredness, restlessness, sleeping problems
Perception with pain	Shoulder pain, pain syndrome, headache
Perception other than pain	Perception and co-ordination deficits, vision or hearing limitation, sensibility impairment, balance disturbance, dizziness
Psycho-social	Inability to control emotions, pathological crying, anxiety, uneasiness, stressfulness, social deprivation, personality change, uncertainty, irritability, depression
Well-being	Deterioration, stroke recurrence
Composite assessment	Other health problems

#### Register data

Mortality and hospital admission data covering the first post-stroke year were obtained from the national Cause of Death Register, covering all deaths among Swedish residents, and the Hospital Discharge Register, covering all hospital admissions in Sweden.

### Ethical considerations

All subjects in the study population gave oral informed consent to participate, the standard procedure at the time. The study was approved on several occasions during the planning and data collection, first by the Research Ethics Committee at Uppsala University and later by the National Research Ethics Board.

### Statistical considerations

Data were analysed using the SAS software, version 9.2 (23). Survival, health care utilization, and diagnoses were 100% complete, and post-morbid state data, based on interviews, were 91% complete.

Functional ability and self-rated health were ordinal variables and were therefore tested with ordinal logistic regression. MMSE, HAD, and NHP were continuous but moderately skewed towards high or low values. They were tested with linear regression based on original data, linear regression based on log-transformed data, and ordinal logistic regression based on grouped data. All methods gave similar results, and therefore only results based on ordinal logistic regression are shown. To obtain the levels of these variables, functional ability, MMSE, self-rated health, HAD, and NHP were computed with the least square mean function of the SAS procedure General Linear Model (linear regression), adjusting for the influence of age, sex, marital status, stroke number, loneliness, and education (covariates).

The prevalence of the VIPS key words based on interview data was analysed with nominal logistic regression, with the key word entered as dependent variable, and time and the covariates as independent variables, providing *P* values for trends across time. The same procedure was used for patient record data computed with the same time-frame as the interview data.

The record-based VIPS key word data were collected continuously on a daily basis. However, for illustration purposes the day-by-day prevalence was transformed to week-by-week prevalence. It was calculated using a set of variables, each variable representing a specific week during the year (week-by-week matrix). Having a specific VIPS key word during a specific week (= 1) or not (= 0) was indicated in the corresponding week's variable. Summing the individual matrices and dividing the variable for each week by the number of exposed patients produced the weekly proportion of patients with a given VIPS key word, adjusted for non-exposure (being in hospital or deceased). Logistic regression was used to test the prevalence change across the year.

Simultaneous occurrence, or co-occurrence, of more than one VIPS key word per patient per measurement occasion was analysed with the ‘substring’ feature available in the SAS software and using factor analysis with varimax oblique rotation. Both methods yielded the same results. Only two-tailed tests were used. To account for the many tests performed, *P* < 0.005 was regarded as statistically significant.

## Results

### Characteristics of the study population

At the index admission, 75% were admitted for their first ever stroke, and 223 (57%) were women. Mean age was 79.4 years for women and 76.8 years for men, and half of the women and one-third of the men were living alone in their own homes. Forty-seven per cent were treated at a stroke unit, and 57% were transferred to the Department of Geriatrics for further care and recovery, where 13 (3%) of the patients died. The mean number of in-hospital days at the Department of Medicine was 7.4 (median 6, interquartile range 3–9), and at the Department of Geriatrics 30.9 (median 27, interquartile range 14–41). Three-quarters of the patients were discharged back to their homes, while the rest were discharged to assisted accommodation or nursing homes. One week after hospital discharge two-thirds of the patients were able to maintain their personal hygiene and were mobile without assistance, and slightly less than 6 out of 10 could dress independently.

### Change of health situation over time

The levels of functional ability, MMSE, self-rated health, HAD, and NHP were stable across the first post-stroke year after adjustment for the influence of the covariates (Table II). The cumulative period prevalence of the interview-based VIPS key words since discharge or last interview and the patient record-based key words arranged in a similar time-frame are shown in Table III. Generally, the prevalence of the interview-based VIPS key words across all three measurement occasions was higher than the record-based ones, with a few exceptions. Based on interview data 82% reported any VIPS key word at the first, 87% at the second, and 97% at the third interview. The corresponding prevalence based on record data was 43, 55, and 92%.

**Table II.** Functional ability according to Katz Activity of Daily Living, Mini Mental State Examination score, self-rated health, Hospital Anxiety and Depression scale, and Nottingham Health Profile scale as measured on three occasions during the first post-stroke year, after adjustment for the influence of age, sex, marital status, stroke number, loneliness, and education.

	Time of measurement			
	1 week after discharge	3 months after index admission	12 months after index admission	*P* for trend
Functional ability (1–7)	2.4	2.2	2.3	0.73
Mini Mental State Examination (0–30)	22.3	24.0	22.6	0.50
Self-rated health (1–5)	2.4	2.3	2.2	0.12
Hospital Anxiety and Depression scale				
Depression (0–21)	4.3	4.3	4.2	0.37
Anxiety (0–21)	3.7	3.3	3.3	0.09
Nottingham Health Profile				
Energy (0–100)	38.2	36.4	36.4	0.97
Physical mobility (0–100)	32.5	30.3	33.4	0.45
Sleep (0–100)	23.1	22.1	22.8	0.75
Emotional reactions (0–100)	20.4	20.4	18.4	0.19
Social isolation (0–100)	18.0	16.6	18.1	0.63
Pain (0–100)	9.6	10.0	11.2	0.51

**Table III.** Period prevalence of health problems according to Well-being, Integrity, Prevention, and Safety (VIPS) classification key words as reported at interview and found in primary health care and municipal elderly health care records, measured on three occasions during the first post-stroke year, after adjustment for the influence of age, sex, marital status, stroke number, loneliness, education, and for non-exposure (hospital admissions and mortality).

		Health problems reported at interviews				Health problems in patient records			
		1 week after discharge	3 months after index	12 months after index	*P* for trend	1 week after discharge	3 months after index	12 months after index	*P* for trend
Health problems, total, %		82.2	87.0	97.2	< 0.0001	43.3	55.2	91.5	< 0.0001
	Perception	47.3	51.5	69.6	< 0.0001	9.7	13.2	42.4	< 0.0001
	Activity	27.9	33.9	64.2	< 0.0001	9.6	12.5	35.3	< 0.0001
	Sleep	36.0	40.6	62.4	< 0.0001	10.9	14.4	41.3	< 0.0001
	Cognition	19.8	23.2	42.5	< 0.0001	2.2	2.9	11.0	< 0.0001
	Pain	19.9	21.8	32.2	< 0.0005	12.0	16.5	50.6	< 0.0001
	Elimination	13.6	16.1	32.1	< 0.0001	19.0	23.4	50.4	< 0.0001
	Nutrition	14.2	16.4	29.3	< 0.0001	6.0	7.9	25.4	< 0.0001
	Breathing or circulation	8.6	10.6	25.4	< 0.0001	7.9	11.3	44.3	< 0.0001
	Communication	12.8	14.5	24.9	< 0.0001	3.5	4.2	8.7	= 0.005
	Psycho-social	12.2	13.4	20.4	< 0.0001	8.2	10.5	28.6	< 0.0001
	Skin	1.6	2.1	6.9	< 0.0001	6.1	8.5	32.2	< 0.0001
	Miscellaneous	2.9	3.5	8.5	< 0.0005	1.3	1.9	10.3	< 0.0001

**Figure 2.**
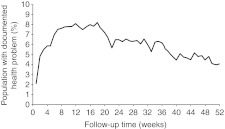
The prevalence of all health problems combined, based on primary health care and municipal elderly health care records during the first post-stroke year, adjusted for non-exposure (hospital admissions and mortality), week by week.

To obtain a better view of the timing of problem-reporting, VIPS key word week prevalence based on record scrutiny over time is shown in Figure 2. The total VIPS key word prevalence increased as the patients were discharged from hospital, peaked at 8% 12–18 weeks after index admission, and then gradually decreased to 4%. All individual key words followed the same pattern, except ‘Skin’ problems, which were fairly constant from week 12 and onwards.

**Figure 3.**
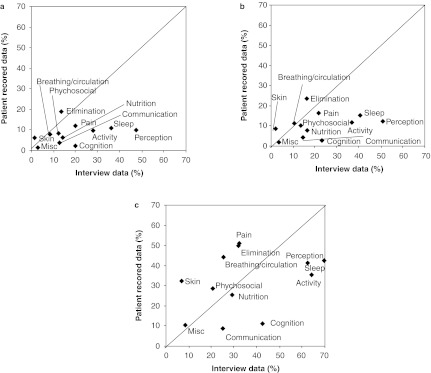
Health problem reporting across time with VIPS key word prevalence based on patient interviews in relation to those based on record scrutiny at 1 week after discharge (a), and 3 (b) and 12 months (c) after index admission. Misc = Miscellaneous.

In Figure 3, interview VIPS key word prevalence is plotted against prevalence based on record scrutiny. The most frequently reported VIPS key words from the interviews were ‘Perception’, ‘Activity’, ‘Sleep’, and ‘Cognition’, while the most common findings based on records were ‘Pain’, ‘Elimination’, ‘Breathing or circulation’, ‘Perception’, and ‘Sleep’. The interview-based key words were more prevalent than the record-based ones at 1 week and 3 months, but at 12 months the prevalence difference was less pronounced.

Based on interviews there was co-occurrence between the VIPS key words ‘Cognition’, ‘Activity’, ‘Sleep’, ‘Pain’, ‘Perception’, and ‘Psycho-social’. A total of 538 (61.6%) interviews contained various combinations of these key words. Other combinations were infrequent. Based on patient record data there was similar co-occurrence between the VIPS key words ‘Breathing or circulation’, ‘Elimination’, ‘Sleep’, ‘Pain’, ‘Perception’, and ‘Psycho-social’. A total of 3,167 (54.1%) patient record notes contained various combinations of these key words.

## Discussion

Functional ability, MMSE, self-rated health, degree of depression, anxiety, and NHP did not change during the first post-stroke year. The rank order of health problems based on patient interviews and patient record scrutiny differed somewhat. The total prevalence of record-based health problems was moderate, peaked in the early part of the first post-stroke year, and then declined substantially.

Strengths of this study included that the study population comprised all patients in the area fulfilling the inclusion criteria and who survived the acute stroke phase. The population was large enough for the purpose of the study, representing more than 90% of the total stroke population, regardless of inclusion criteria, during the recruitment period (14). The data were collected with validity- and reliability-tested instruments. The documentation of health problems in the patient interviews and the complete set of hospital and PHCC patient records and municipality elderly health care records during the first post-stroke year were performed in such a way that all health problems mentioned or noted were recorded. The patient record data covered notes from physicians as well as nurses, occupational therapists, and physiotherapists. Most of the notes were nursing documentation. The PHCC records were computerized, which meant that notes had to be included in a standardized format.

The limitations of this study include that the patients in the interviews were instructed to report problems occurring after a given point in time (the day of discharge in the first interview, or the previous interview in the second and third). In addition to memory problems the patients may have had difficulties in differentiating problems occurring before and after the specified point in time, or may even have reported cumulative data across the year. However, the information obtained from the interviews gave supplementary information to that obtained from patient records. Another limitation might be the fact that no control group of stroke-free patients was available to allow for separation of stroke-related health problems from other problems.

A large number of health problems were recorded. Sometimes presumably identical problems were worded in different ways, and some problem wording overlapped. In order to facilitate the understanding and analysis of the health problems, the VIPS classification was adopted. VIPS was originally created and validity-tested to allow for systematic nursing documentation (22), but it also proved to be suitable for our classification of health problems in patient records. Of the original 15 VIPS key words, ‘Sexuality’ and ‘Spiritual/cultural’ were not used in the present study, and ‘Composite assessment’ and ‘Well-being’ were amalgamated to ‘Miscellaneous’.

As far as we know the continuous serial measurements used for patient record data in this study have not been used before. Serial measurements, resembling our interview data collection technique, have been used by others for various stroke-related disability outcomes, such as depression (3,6,12), anxiety (6), function (3), falls (4,6) and fractures (4,11), fatigue (9,24), cognition (3), dementia (25), and pain (4,6). Whether or not the depression or anxiety prevalence changes during the first post-stroke year is controversial, but it has been suggested that depression may vary over time, confirming its dynamic nature (26).

All but one of the record-based health problems analysed in this study had the same time-course as the total health problem prevalence presented in Figure 2. There may be a number of explanations for the decrease in prevalence over time. It may be attributable to registration bias, if long-term health problems are no longer reported, although since all but one health problem followed the same course across time this alternative seems unlikely. A second possibility might be that the health problems were solved or healed. A third possibility could be that the decline might be attributable to selective mortality, the most severely ill people being those who died during follow-up. However, in the analyses each subject was compared with him- or herself longitudinally by means of adjustments made (use of covariates), making this possibility less likely.

We found reporting differences between health problems based on interviews and those based on patient records (Figure 3). The completeness of nursing documentation has been studied in a Danish hospital. Of all nursing problems known, one-third was documented, another third was known by the staff but not documented, and one-third was known only by the patients (27). Others have found that patients identify severe problems that are unknown to the nursing staff (27–29), and that there is inadequate staff ability to identify unmet needs (30). In this study the view of both parties was taken into account. These discrepancies in reporting may be attributable to different views of health problems. For instance, ‘Pain’, ‘Elimination’, and ‘Breathing or circulation’ problems may be effectively handled by health care staff, and they may not be very annoying to the patient. On the other hand, ‘Perceptive’ and ‘Cognitive’ problems are not easily communicated, and unvoiced problems will not be addressed (31). Neither the patient nor the health care professionals may have the words or the tools to handle these problems, and the professionals may not ask about problems for which they do not have a solution. Furthermore, vague symptoms such as tiredness, sleep problems, low mood, and forgetfulness are often considered by patients (32) and health care staff to be normal in old age.

There are three lessons to be learnt from this study. First, the situation during the first post-stroke year may not be as bad as previously thought. The cumulative reporting method, usually used in studies similar to the present one, appears to indicate that the level of health problems is high. The alternative method used in this study, a continuous time series of health problems as reported in patient records, provides a more straightforward estimate of health problem prevalence. In this comparative study the cumulative method indicated health problems in more than 90% of the study population versus 8% or less using the continuous time series method. It should be pointed out that the results given in Table III are cumulative period prevalence data covering a fairly long time period, whereas the results in Figure 2 are week prevalence based on a week-by-week matrix. The reason for the difference at the time of the first interview, 43% versus 8%, is the wide range of days in hospital, the first interview being held 5–185 days after the index hospital admission; with the serial method this time difference could be controlled for, but not with the cumulative method. The cumulative method probably overestimates the health problem level, whereas the continuous serial method may underestimate it.

The second lesson is that the health care organization may be efficient and effective when dealing with concrete ‘hands-on’ problems, but may lack the corresponding skill in identifying more subtle problems, such as cognitive or communication problems. Such problems cannot easily be treated or affected, but should nonetheless be identified.

The third lesson concerns co-occurrence of health problems. In this study we found frequent co-occurrence between ‘Cognition’, ‘Activity’, ‘Sleep’, ‘Pain’, ‘Perception», ‘Psycho-social’, ‘Breathing and circulation’, and ‘Elimination’ health problems. Appelros et al. (10) found similar co-occurrence. Detection of one of these health problems should lead to consideration of the others. The awareness of this co-occurrence may help health care professionals in their assessment of stroke patients' health status.

## Conclusions

In conclusion, most stroke survivors had some health problem during the first year, but only 4–8% were reported in records during a given week. The health problems peaked early after discharge and then declined. There were differences in prevalence rates between interview and record data, with the main discrepancies regarding perception, cognition, and communication. There was co-occurrence between some of the health problems, which might be used to identify unvoiced health problems.
